# Expression of the BAD pathway is a marker of triple-negative status and poor outcome

**DOI:** 10.1038/s41598-019-53695-0

**Published:** 2019-11-25

**Authors:** Bernadette M. Boac, Forough Abbasi, Roohi Ismail-Khan, Yin Xiong, Atif Siddique, Hannah Park, Mingda Han, Daryoush Saeed-Vafa, Hatem Soliman, Brendon Henry, M. Juliana Pena, E. Clair McClung, Sharon E. Robertson, Sarah L. Todd, Alex Lopez, Weihong Sun, Susmitha Apuri, Johnathan M. Lancaster, Anders E. Berglund, Anthony M. Magliocco, Douglas C. Marchion

**Affiliations:** 1 Department of Anatomic Pathology, H. Lee Moffitt Cancer Center and Research Institute, Tampa, FL 33612 USA; 2 Chemical Biology and Molecular Medicine, H. Lee Moffitt Cancer Center and Research Institute, Tampa, FL 33612 USA; 3 Cedars-Sinai Medical Center, Los Angeles, CA 90048 USA; 4 Department of Oncologic Sciences, H. Lee Moffitt Cancer Center and Research Institute, Tampa, FL 33612 USA; 5 Department of Women’s Oncology, H. Lee Moffitt Cancer Center and Research Institute, Tampa, FL 33612 USA; 6 University of Arizona Cancer Center, Obstetrics and Gynecology, Tucson, AZ 85724 USA; 7 Indiana University Health, Indianapolis, IN 46202 USA; 8 Myriad Genetics, Salt Lake City, UT 84108 USA; 9 Department of Bioinformatics and Biostatistics, H. Lee Moffitt Cancer Center and Research Institute, Tampa, FL 33612 USA; 10 Protean Diagnostics, Tampa, FL 33612 USA

**Keywords:** Preclinical research, Breast cancer, Targeted therapies

## Abstract

Triple-negative breast cancer (TNBC) has few therapeutic targets, making nonspecific chemotherapy the main treatment. Therapies enhancing cancer cell sensitivity to cytotoxic agents could significantly improve patient outcomes. A BCL2-associated agonist of cell death (BAD) pathway gene expression signature (BPGES) was derived using principal component analysis (PCA) and evaluated for associations with the TNBC phenotype and clinical outcomes. Immunohistochemistry was used to determine the relative expression levels of phospho-BAD isoforms in tumour samples. Cell survival assays evaluated the effects of BAD pathway inhibition on chemo-sensitivity. BPGES score was associated with TNBC status and overall survival (OS) in breast cancer samples of the Moffitt Total Cancer Care dataset and The Cancer Genome Atlas (TCGA). TNBC tumours were enriched for the expression of phospho-BAD isoforms. Further, the BPGES was associated with TNBC status in breast cancer cell lines of the Cancer Cell Line Encyclopedia (CCLE). Targeted inhibition of kinases known to phosphorylate BAD protein resulted in increased sensitivity to platinum agents in TNBC cell lines compared to non-TNBC cell lines. The BAD pathway is associated with triple-negative status and OS. TNBC tumours were enriched for the expression of phosphorylated BAD protein compared to non-TNBC tumours. These findings suggest that the BAD pathway it is an important determinant of TNBC clinical outcomes.

## Introduction

Approximately 17% of the 1 million cases of breast cancer diagnosed annually worldwide are of the triple-negative (oestrogen receptor– (ER-)/progesterone receptor– (PR-)/HER2-negative) phenotype^1^. Triple-negative breast cancer (TNBC) is biologically aggressive and disproportionately affects premenopausal women, with the highest prevalence found among women of African and Hispanic descents^2–5^. African-American, Hispanic, and Caucasian women account for approximately 47%, 23%, and 15% of TNBC cases, respectively^5,6^. After adjusting for age and stage at diagnosis, African-American women are almost 3 times more likely than Caucasian women to have TNBC^6^.

Few targeted agents are available for the treatment of TNBC tumours. The standard of care for patients with TNBC is nonspecific chemotherapeutic agents, such as those that induce DNA damage (ie, platinums, anthracyclines) or microtubule destabilizations (paclitaxel, taxotere)^7^. Although TNBC tumours are initially sensitive to standard chemotherapy, patients with this disease are at greater risk of recurrence and typically experience a very short disease-free survival span^6,8,9^. Among patients who have incomplete responses to chemotherapy, those with TNBC experience worse overall survival (OS) outcomes than those with non-TNBC^10^. To date, no targeted agents have shown broad effectiveness against TNBC. Although polyADP-ribose polymerase inhibitors have shown some efficacy, the effectiveness of these targeted agents is limited to relatively small subsets of TNBC patients^11–13^ therefore, there is a critical need for more active therapeutic strategies.

BCL2 antagonist of cell death (BAD) is a member of the BCL2 family of proteins, which includes inhibitors and promoters of apoptosis, such that cell survival is determined by the relative ratio of proapoptotic (e.g., BIM, BAD, BAX, BAK) and antiapoptotic (e.g., BCL-2, BCL-xL, MCL-1, A1, BAG-1) family members^14–17^. BAD selectively hetero-dimerizes with BCL-xL and BCL-2 but not with BAX, BCL-xs, MCL-1, A1, or itself. When BAD dimerizes with BCL-xL, BAX is displaced, mitochondrial membrane permeability increases, and apoptosis is induced^18^. BAD function is regulated by phosphorylation (including serine −112, −136, and −155)^19–21^. When phosphorylated, BAD is unable to heterodimerize with BCL-2 or BCL-xL, which frees BCL-xL to dimerize and functionally sequestrate BAX, resulting in BAX no longer being free to induce apoptosis^18^. Thus, BAD phosphorylation determines whether BAX is displaced from BCL-xL to drive cell death. BAD is phosphorylated at serine-136 by protein kinase B (PKB/AKT)^22^. In contrast, serine-112 of BAD is phosphorylated by mitogen-activated protein kinase–activated protein kinase-1 (MAPKAP-K1, also called RSK) and protein kinase A (PKA). Serine-155 at the centre of the BAD BH3 domain is phosphorylated preferentially by PKA, which also inhibits BCL-xL binding^21,23,24^. Conversely, the activity of a series of phosphatases, including PP1, PP2A, and PPM1 (PP2C/PPM1A), as well as calcineurin, has been shown to have proapoptotic effects via dephosphorylation of BAD^25,26^.

We previously reported that the expression of the BAD pathway and levels of phosphorylated BAD protein were associated with chemoresistance in ovarian cancer^27–29^. Due to the reported molecular similarities between ovarian cancer and TNBC^30–32^, we evaluated the influence of BAD pathway expression on chemoresponse in TNBC. In this report, we show that the BAD pathway expression can differentiate between triple-negative phenotype breast cancers and those that express ER and/or PR in tumours and cell lines; furthermore, the BAD pathway expression was found to be associated with OS in 2 independent clinico-genomic datasets. We also show that, compared to non-TNBC cases, TNBC tumours were enriched in the expression of phosphorylated BAD isoforms. We demonstrated *in vitro* that targeted inhibition of kinases known to phosphorylate BAD protein sensitized TNBC cells but not ER-/PR-positive cells to the cytotoxic effects of cisplatin.

## Materials and Methods

### Patients

This study was performed as part of a University of South Florida IRB–approved protocol and in accordance with the relevant guidelines and regulations, including Code of Federal Regulations Title 45 Part 46 Protection of Human Subjects. Following IRB approval, patient samples and molecular and clinical data stored in the Moffitt Cancer Center (MCC) Total Cancer Care (TCC) clinico-genomic tissue and data repository were accessed (MCC 14690/Liberty IRB #Pro00014441). All patients whose samples and data are included in the TCC protocol have provided prospective written informed consent for their use in research. Breast cancer samples from TCC were limited to those with complete clinical information and Affymetrix gene expression data. To ensure balance within the samples, only breast cancer patients whose carcinomas *did not* express HER2 receptors were included. Using these criteria, samples from 53 non-TNBC and 53 TNBC patients were available in the TCC database and analysed in this study. Chart abstractions were used to collect the following clinical elements: age, stage, grade, body mass index (BMI), gravida, tumour size, surgery, lymph-node status, and OS. The non-TNBC and TNBC groups were well balanced with no significant differences in age (non-TNBC, mean = 51.84 ± 1.65; TNBC, mean = 52.34 ± 1.55; *t* test *P* = 0.83), stage (chi-square test, *P* = 0.33), tumour size (non-TNBC, 2.95 ± 0.24; TNBC, 2.65 ± 0.25; *t* test *P* = 0.38), or BMI (non-TNBC, 28.32 ± 0.88; TNBC, 28.76 ± 1.1; *t* test *P* = 0.76); however, the TNBC group included more patients with grade 3 disease (non-TNBC, 38%; TNBC, 83%; chi-square test, *P* = 2.55 × 10^−5^).

### RNA extraction and microarray expression analyses

In accordance with TCC protocol, all tissues were snap frozen within 15 minutes of collection, macro-dissected to ensure >80% tumour content, and quantified for the percentage of malignancy, cellularity, stroma, normalcy, and necrosis. Approximately 30 mg of tissue for each sample were pulverized in BioPulverizer H tubes (Bio101) using a Mini-Beadbeater (Biospec Products). Total RNA was collected using the Qiagen RNeasy Mini kit in accordance with manufacturer’s instructions. An Agilent Bioanalyzer was used to assess RNA quality via the 28S:18S ribosomal RNAs. Ten micrograms of total RNA were used to develop the targets for Affymetrix microarray analysis, and probes were prepared according to the manufacturer’s instructions. Briefly, biotin-labelled cRNA was produced by *in vitro* transcription, fragmented, and hybridized to customized Human Affymetrix HuRSTA gene chips (HuRSTA-2a520709). Expression values were calculated using the robust multi-array average algorithm implemented in Bioconductor (http://www.bioconductor.org) extensions to the R statistical programming environment. The gene expression data discussed in this publication have been deposited in National Center for Biotechnology Information’s Gene Expression Omnibus (GEO) and are accessible through GEO series accession number GSE62931^33^.

### Deriving a BAD pathway Principal components analysis score

The BAD pathway gene expression signature (BPGES) was developed from the GeneGo Metacore–defined BAD Apoptosis and Survival Pathway using the genes that showed importance in the PCA model. These included BAX, BCL2, EGFR, PDK1, PIK3CA, PIK3CB, PPP1CA, PPP2CA, PPP3CA, PPM1A, YWHAB, YWHAE, YWHAG, YWHAH, YWHAQ, and YWHAZ. All genes in the BPGES have previously been shown to directly or indirectly influence the phosphorylation status and/or apoptotic activity of BAD protein; these are BAX^34,35^, BCL-2, EGFR^36,37^, PDK1 (PDPK1)^38^, PI3 kinase (PIK3CA, PIK3CB)^36^, PP1 (PPP1CA)^39^, PP2A (PPP2CA)^40^, Calcineurin (PPP3CA)^41^, (PP2C) PPM1A^25,27,42^, and 14.3.3 (YWHAB, YWHAE, YWHAG, YWHAH, YWHAQ, YWHAZ)^43^. The PCA methodology was used to derive a BPGES pathway score that would represent overall gene expression levels for these BAD-pathway genes. Genes and probe sets used in the PCA model for the different datasets are listed in the Supplemental Table S1. Only 1 probe set was used per gene, which was selected on the basis of the highest expression value in the TCC dataset samples.

PCA is a well-established technique for unsupervised data analyses and dimensional reduction, as described by Joliffe and Ma^44,45^. We and others have previously shown that the first component of a PCA model, defined as PC1, can successfully compare the expression of gene signatures and describe pathway activation in tumour samples. It can also be used for survival analyses^29,38^. In brief, the first step when using PCA to compare signature expression in clinico-genomic datasets is to create a subdataset by selecting only the probesets in the given gene expression signature. To calculate the BPGES, probesets representing 16 genes within the BAD pathway were reduced to a set of uncorrelated principal components. After removing the column mean (mean centring) and scaling each column-to-unit variance, the PC1 score can be calculated. That is, the pathway score is ∑w_*i*_x_*i*_, a weighted-average expression among the BAD-pathway genes in which x_*i*_ represents gene *i* expression level, w_*i*_ is the corresponding weight (loading coefficient) with ∑w^2^_*i*_ = 1, and the w_*i*_ values maximize the variance of ∑w_*i*_x_*i*_. PC1 describes the direction in the N-dimensional space (in which N is the number of genes) that maximizes the explained variance. By not using all the variation in the data, PC1 is stable to random noise, can handle missing values, and provides a simple score for each sample. Thus, the PC1 score is a numeric value that summarizes the expression pattern of the entire signature and represents an overall similarity measurement between samples on the basis of their expression profile for the selected genes. The corresponding loadings are related to the variables (e.g., Affymetrix probe sets) and reflect relative importance in the PCA model.

### Statistical considerations

Principal components analysis (PCA) was used to derive the BPGES. Differential expression of BPGES between groups was determined using Student’s *t* test analyses of derived PCA scores. Log-rank (Mantel-Cox) significance tests of Kaplan-Meier curves were used to compare the associations of BPGES PCA scores and phospho-BAD protein expression scores with OS. OS was defined as the period from the date of diagnosis to the date of death from any cause. Median OS was used to define the high-versus-low BPGES. Pearson’s correlation test was used to evaluate the correlations between phospho-BAD isoform expression scores and other phospho-BAD isoforms as well as the BPGES. The Mann-Whitney test and unpaired *t* tests were used to compare the expression of individual genes within datasets and the BPGES between breast cancer subtypes.

### External datasets

The BPGES was evaluated in breast cancer samples and cell lines from publicly available datasets, including 1) The Cancer Genome Atlas (TCGA [https://cancergenome.nig.gov/]; n = 757 breast cancer samples [84 TNBC, 324 hormone receptor positive, 349 HER2 positive] with RNAseq data); 2) Cancer Cell Line Encyclopedia (CCLE [https://software.broadinstitute.org/software/cprg/?q=node/11]; n = 42 breast cancer cell lines [14 non-TNBC, 28 TNBC] with Affymetrix U133-Plus 2.0 expression data). For the CCLE data, raw cell file expression data were processed in R using the Affymetrix Expression Console.

### Immunohistochemistry staining and histologic scoring

Formalin-fixed, paraffin-embedded samples were used to produce a tissue microarray (TMA) comprised of 3 replicate cores (0.6 mm each) of 36 TNBC samples and 18 non-TNBC samples, as well as duplicate cores of control tissues and staining controls. Case selection for the TMA was based on the availability of gene expression data, clinical information, and tissue. The TMA was evaluated by immunohistochemistry (IHC) for the expression of phospho-BAD-serine-112 (Ser^112^, A00295, Genscript, Atlanta, GA, USA, 1:800 dilution), phospho-BAD-serine-136 (Ser^136^; A01156, Genscript, 1:200 dilution), and phospho-BAD-serine-155 (Ser^155^; AB28825, Abcam, Cambridge, MA, USA, 1:50 dilution). BAD-Ser^112^, -Ser^136^, and –Ser^155^ are mouse BAD protein designations and are analogous to human BAD-Ser^75^, -Ser^99^, and –Ser^118^, respectively^29,46,47^. Antibodies recognizing these phosphorylated serine residues are cross reactive between human and mouse BAD protein. For simplicity, only the mouse designations are used in this manuscript. Before samples were stained, they were deparaffinised and rehydrated using xylene, followed by serial dilutions of ethanol. Slides were stained using a Ventana Discovery XT automated system (Ventana Medical Systems, Tucson, AZ, USA) with proprietary reagents, in accordance with the manufacturer’s protocol. Briefly, slides were deparaffinised on the automated system with EZ Prep solution (Ventana). A heat-induced antigen retrieval method was used in Cell Conditioning 1 (Ventana). Primary antibody optimization and tissue staining was performed under the direction of the study pathologist using manufacturer-suggested methods and control tissues. Antibodies were diluted in PSS antibody diluent (Ventana), incubated for 60 minutes at room temperature, rinsed with diluent, and incubated with the appropriate horse radish peroxidase- (HRP-) linked secondary antibodies for 16 minutes. Antibody binding was detected using the Ventana ChromoMap kit, and slides were counterstained with haematoxylin. Slides were then dehydrated and coverslipped in accordance with normal laboratory protocol. To compare phospho-BAD isoform levels, expression scores were determined by the study pathologist using the product of intensity and cellularity, in which an intensity of 1 was considered weak, 2 moderate, and 3 strong, and a cellularity of 1 was ≤33%, 2 was 34% to 65%, and 3 was ≥66%^48,49^. In addition, Definiens software was used to generate a histo-expression score (H-score). Stained TMA slides were scanned using the Aperio (Vista, CA, USA) AT2 with a 200x/0.8NA objective lens at a rate of 3 minutes per slide via Basler trilinear-array detection. Each core was then segmented for individual analyses using Spectrum’s TMA block software. Image analyses were performed to segment positive staining of various intensities using an Aperio Positive Pixel Count v9.0 algorithm with the following thresholds: hue value = 0.1, hue width = 0.5, colour saturation threshold = 0.04, intensity weak positive (high) = 220, intensity weak positive (low) = intensity positive (high) = 175, intensity positive (low) = intensity strong positive (high) = 100, and intensity strong positive (low) = 0. The algorithm was applied to the entire digital core image to determine the percentage of positive biomarker staining by applicable area. Tissue cores on the TMA were manually inspected by the study pathologist and removed from the analysis when less than 50% of the tissue was remaining, tissue folds were evident, or obvious artefacts were present.

### Cell culture and reagents

All cell lines were obtained from the American Type Culture Collection (Manassas, VA, USA) and cultured in RPMI (MDA-MB-157, MDA-MB-436, BT549, and Hs578t) supplemented with 10% foetal bovine serum, 1% sodium pyruvate, and 1% nonessential amino acids or DMEM (MCF-7, T47D, MDA-MB-231, and MDA-MB-468) supplemented with 10% foetal bovine serum. All media were supplemented with Mycozap Plus-CL (Lonza, Rockland, ME, USA) for the prevention of mycoplasma and bacterial contamination. All tissue culture reagents were obtained from Fisher Scientific (Pittsburgh, PA, USA). Mycoplasma testing was performed every 6 months in accordance with the manufacturer’s protocol (Lonza). The AKT inhibitor perifosine was acquired from SelleckChem (S1037; Houston, TX, USA). The PKA inhibitor H89-dihydrochloride Hydrate (H89) was purchased from Sigma-Aldrich (SKU 19–141; St Louis, MO, USA). Both inhibitors were solubilized in dimethyl sulfoxide at a stock concentration of 20 mM and stored at −20 °C until use.

### Western blot analysis

Cells were harvested in media using a Cell Lifter (Thermo Fisher Scientific, Pittsburgh, PA, USA) and washed with cold phosphate-buffered saline containing 1x phosphatase inhibitor cocktail (Sigma-Aldrich, St. Louis, MO, USA). Lysates were prepared with sodium dodecyl sulphate (SDS) lysis buffer (2% SDS, 10% glycerol, 0.06 M Tris, pH 6.8) and evaluated for protein concentration using the bicinchoninic acid method (Pierce, Rockford, IL, USA). Proteins (50–100 μg) were separated on the same day as collection on 12% to 15% SDS-polyacrylamide gel electrophoresis gels and transferred to polyvinylidene fluoride membranes. Membranes were blocked with 5% nonfat milk in Tris-buffered saline containing 0.05% Tween 20 (TBST) and incubated with primary antibody in SuperBlock blocking buffer (Thermo Fisher Scientific) with 0.05% Tween 20 overnight at 4 °C. Membranes were washed 3 times for 5 minutes with TBST and incubated with the appropriate secondary antibody in 5% nonfat milk in TBST for 60 minutes at room temperature. Membranes were washed 4 times for 5 minutes with TBST prior to antibody-binding visualization by Super Signal West Pico chemiluminescence solution (Pierce) on autoradiography film (Midwest Scientific, St. Louis, MO, USA). Densitometry analysis was performed on scanned TIFF files using ImageQuant 5.2 software. Antibodies used for the Western blot experiments included phospho-AKT-Ser^473^ (#4060, Cell Signaling Technology, 1:500 dilution), AKT (#4691, Cell Signaling Technology, 1:500 dilution), phospho-PKA (#5661, Cell Signaling Technology, 1:500 dilution), PKA (#4782, Cell Signaling Technology, 1:500 dilution), and GAPDH (#MAB374, Millipore, Billerica, MA, USA 1:2000 dilution). Relative protein levels were determined by densitometry analysis of scanned Western blot film TIFF images from triplicate experiments using Alpha View software. Full Western blot images for all markers can be viewed in online Supplementary Fig. S5.

### Cell viability assays

The CellTiter96 MTS assay kit (Promega, Madison, WI, USA) was used to assess cancer cell survival in the presence of the AKT inhibitor perifosine and the PKA inhibitor H89 dihydrochloride hydrate^50–53^. For the assays, 3 to 5 × 10^4^ cells in 100 µL were plated to each well of a 96-well plate and allowed to adhere overnight at 37 °C and 5% CO_2_. The following day, cells were incubated with increasing concentrations of the chemotherapeutic agent for 72 hours. Three replicate wells were used for each drug concentration, and an additional 3 control wells received a diluent control without drug. One well (blank) was devoid of cell and was used to assess the background optical density readings of media and reagents. After drug incubation, the optical density of each well was read at 490 nm using a SpectraMax 190 microplate reader (Molecular Devices Inc., Sunnyvale, CA, USA). Percent cell survival was expressed as control-treated/control-blank × 100. All experiments were performed at least 3 times to ensure reproducibility and accuracy of the results. To compare sensitivities, IC_50_ values for cisplatin and cisplatin and perifosine or H89 hydrate were computed using sigmoidal dose-response algorithm implemented in GraphPad (GraphPad Software, Inc, San Diego, CA, USA).

### BAD protein depletion

Pooled RNA duplexes for BAD (SMARTpool:On Target Plus BAD siRNA ID#572, Dharmacon/GE Life Sciences, Lafayette, CO, USA target sequence: UCGGAAGUUUUGGGUUUUC) were used to transfect breast cancer cells (4 × 10^6^) using the Nucleofector transfection kit containing Transfection Buffer V (#VCA1003, Lonza), in accordance with manufacturer’s protocols. The nontargeting *Silencer*-negative control #2 small interfering RNA (siRNA; Ambion/ThermoFisher Scientific) was used as a control. Subsequent depletion of BAD protein was determined by Western blot analyses.

## Results

### BAD pathway expression associates with triple-negative status and overall survival

**Figure 1:**
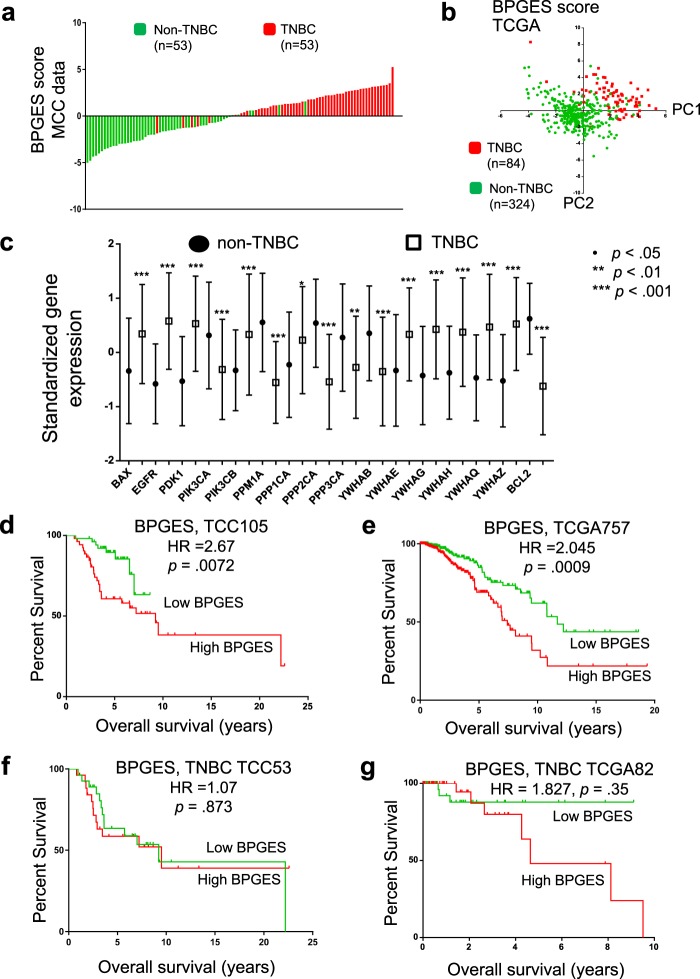
The BAD pathway gene expression signature (BPGES) was associated with triple-negative status and breast cancer survival. **(a)** Waterfall plot showing differential expression of the BPGES in non-TNBC samples (green, *n* = 53, mean BPGES = −1.7 ± 0.24) and TNBC samples (red, *n* = 53, mean BPGES = −1.7 ± 0.19) in the TCC breast cancer dataset (*p* = 4.8 × 10^−19^). **(b)** Scatter plot showing clustering of non-TNBC samples (green, *n* = 324) separate from TNBC samples (red, n = 84), when the first principal component (PC1) of the PCA model is graphed against the second principal component (PC2) in TCGA breast cancer samples. **(c)** Boxplots showing the differential expression of individual genes of the BPGES between non-TNBC (solid circles) and TNBC cases (open squares) from the TCC dataset. Unpaired T-test indicates significance of ***p < 0.001, ** p < 0.01, *p < 0.05, no symbol: p > 0.05. Kaplan-Meier curves showing the association between high BPGES (median threshold) and reduced OS for non-TNBC and TNBC patients in the (d) TCC dataset (*n* = 105, log-rank test, hazard ratio (HR) = 2.67, *p* = 0.0072), (**e**) TCGA dataset (*n* = 757, log rank test, HR = 2.045, *p* = 0.0009) and only TNBC patients of the (**f**) TCC dataset (*n* = 53, log rant test, HR = 1.07, *p* = 0.873) a*n*d (**g**) TCGA dataset (*n* = 82, log rant test, HR = 1.827, *p* = 0.35).

To evaluate the overall expression and activation of the BAD pathway in breast cancer, PCA was used to generate a BPGES score. The genes comprising the BPGES included BAX, BCL-2, EGFR, PDK1, PIK3CA, PIK3CB, PPP1CA, PPP2CA, PPP3CA, PPM1A, YWHAB, YWHAE, YWHAG, YWHAH, YWHAQ, and YWHAZ. PCA modelling of the BPGES in a gene expression dataset, comprised of 106 breast cancer patient samples (53 non-TNBC and 53 TNBC), indicated that a high BPGES score was associated with breast cancers displaying the triple-negative phenotype, with a mean BPGES score of −1.7 ± 0.24 for non-TNBC and 1.7 ± 0.19 for TNBC (unpaired *t* test, *P* = 4.8 × 10^−19^; Fig. 1a). As the TNBC group contained a significantly higher percentage of grade 3 tumours, we evaluated whether the BPGES was associated with disease grade. The mean BPGES score was 1.46 ± 0.58 for low-grade disease (grades 1 and 2) versus 1.92 ± 0.24 for high-grade disease (grades 3 and 4; *t* test, *P* = 0.4973); therefore, it was not associated with grade of disease. The association between the BPGES and triple-negative status was confirmed by using hormone receptor positive (non-TNBC) and TNBC samples from TCGA (n = 408 [84 TNBC, 324 hormone receptor positive], mean BPGES scores were −0.54 ± 0.08 for non-TNBC and 2.13 ± 0.17 for TNBC [*P* = 1.89 × 10^−26^]; Fig. 1b). As shown in Fig. 1b, PCA modelling of the BPGES showed distinct clustering of TNBC samples separate from non-TNBC samples when the first principal component (PC1) was graphed against the second principal component (PC2). Figure 1c shows the performance of the individual genes of the BPGES in the TCC dataset (n = 106). Similar performance of the BPGES genes was observed in the TCGA dataset (Supplementary Fig. S1).

To determine whether BAD pathway expression influenced patient survival, the BPGES score was evaluated for associations with patient survival in both the TCC and TCGA datasets. Using the median BPGES score as a cut-off to dichotomize samples for analysis, log-rank testing of Kaplan-Meier curves indicated that a high BPGES score was associated decreased OS in the TCC dataset (hazard ratio [HR] = 2.76, *P* = 0.0072; n = 105, survival data available for 52 of 53 non-TNBC) (Fig. 1d) and the TCGA dataset (n = 757, hazard ratio = 2.045, *P* = 0.0009) (Fig. 1e). The BPGES was not associated with OS when only TNBC samples were considered in either the TCC dataset (HR = 1.07, *P* = 0.873, *n* = 52) (Fig. 1f) or the TCGA dataset (HR = 1.827, *P* = 0.35, *n* = 82, survival data available for 82 of 84 TNBC cases) (Fig. 1g). As TNBCs are more aggressive than nonTNBCs, we evaluated the role of the cell cycle in the association between the BPGES and decreased OS among patients with breast cancer. Although we observed a correlation between the BPGES and the Hallmark gene set cell cycle signature (r^2^ = 0.544, *P* < 0.0001) (Supplementary Fig. 1b), the cell cycle signature was not associated with OS in the TCC dataset (HR = 1.721, *P* = 0.1284) (Supplementary Fig. 1c). These data indicate that the cell cycle is an aspect of the BPGES but do not explain the association between the BPGES and OS.

### TNBC cases express higher mean levels of phospho-BAD isoforms than non-TNBC cases

**Figure 2:**
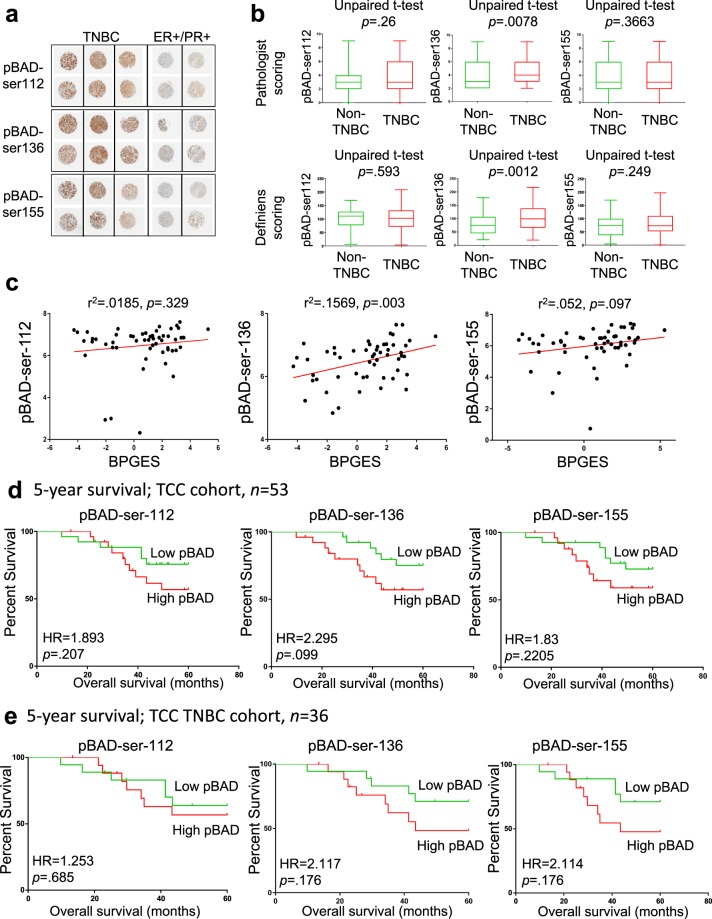
phospho-BAD-Ser^136^ expression is enriched in TNBC and correlates with the BPGES. (**a)** Replicate representative cores of a TMA showing higher levels of phospho-BAD (pBAD) isoforms in TNBC samples when compared to non-TNBC samples. **(b)** Box plots comparing histological expression scores derived from pathologist assessment (top panel) and Definiens software analysis (bottom panel) of phospho-BAD-Ser^112^, phospho-BAD-Ser^136^, and phospho-BAD-Ser^155^ immunohistochemistry stains. Unpaired T-test p-values indicate significance. **(c)** Scatter plots showing the correlations between Definien’s Histo-scores for the phospho-BAD isoforms and the BPGES. Kaplan-Meier, 5-year survival curves showing the association between phospho-BAD isoform expression and decreased survival probability in **(d)** *n* = 54 breast cancer cases (non-TNBC; *n* = 18, and TNBC; *n* = 36) and **(e)** *n* = 36 TNBC cases. Significance was determined using log rank tests. Hazard ratios (HR) show risk. All graphs were generated using GraphPad Prism 7.

To determine the association between the BPGES and the phosphorylation status of BAD protein, a TMA was generated using triplicate cores of 18 non-TNBC and 36 TNBC samples with both gene expression and available tissue. The TMA was evaluated by IHC staining using antibodies specific to phospho-BAD isoforms. Representative core samples for each of the stains are shown in Fig. 2a. Mean histological expression scores (cellularity × intensity) of all cores/cases based on pathologist assessment of IHC staining (scale of 1–3) suggested that TNBCs expressed higher mean levels of phospho-BAD-Ser^136^ (*P* = 0.0078) than non-TNBC samples. Although phospho-BAD-Ser^112^ and phospho-BAD-Ser^155^ also appeared to be higher in TNBC, statistical significance was not reached (phospho-BAD-Ser^112^; *P* = 0.26, phospho-BAD-Ser^155^; *P* = 0.3663, Fig. 2b, top panel). Definiens software–derived Histo-scores of phospho-BAD isoform levels showed similar results. Unpaired *t* tests of Definiens-derived Histo-scores suggested that TNBCs expressed higher mean levels of phospho-BAD-Ser^136^ (*P* = 0.0012) but not phospho-BAD-Ser^112^ (*P* = 0.593) or phospho-BAD-Ser^155^ (*P* = 0.249) (Fig. 2b, lower panel). The TMA Map, pathologist expression scores, Definiens-derived Histo-scores, and IHC stains of the phospho-BAD isoforms can be viewed in Supplemental Tables S2 to S4 and Supplemental Fig. S2 to S5.

Definiens Histo-scores were used for subsequent analyses, given the greater range of expression values. A correlation was observed between Definiens Histo-scores and expression of the BPGES for phospho-BAD-Ser^136^ (r^2^ = 0.1569, *P* = 0.003) but not phospho-BAD-Ser^112^ (r^2^ = 0.0185) or phospho-BAD-Ser^155^ (r^2^ = 0.097) (Fig. 2c). Stronger correlations were observed in the protein levels between the phosphorylated BAD protein isoform levels when all cases (*n* = 54 cases, non-TNBC; *n* = 18, TNBC; *n* = 36) were analysed together (phospho-BAD-ser^112^ versus phospho-BAD-ser^136^; r^2^ = 0.1816, phospho-BAD-ser^112^ versus phospho-BAD-ser^155^, r^2^ = 0.7739; phospho-BAD-ser^136^ versus phospho-BAD-ser^155^; r^2^ = 0.2205) (Supplementary Fig. S6).

Log-rank tests of Kaplan-Meier curves suggested that non-TNBC and TNBC breast cancer patients were at an increased risk of death if their tumours displayed increased expression of pBAD isoforms, although statistical significance was not reached (phospho-BAD-Ser^112^; HR = 1.893, *P* = 0.207, phospho-BAD-Ser^136^; HR = 2.295, *P* = 0.099, phospho-BAD-Ser^155^; HR = 1.83, *P* = 0.2205 (Fig. 2d). A similar trend was observed when only TNBC cases were evaluated; however, statistical significance was not reached (phospho-BAD-Ser^112^; HR = 1.253, *P* = 0.685, phospho-BAD-Ser^136^; hazard ratio = 2.117, *P* = 0.176, phospho-BAD-Ser^155^; HR = 2.114, *P* = 0.176 (Fig. 2e).

### Targeted inhibition of the BAD pathway in TNBC cells potentiates chemotherapy

**Figure 3:**
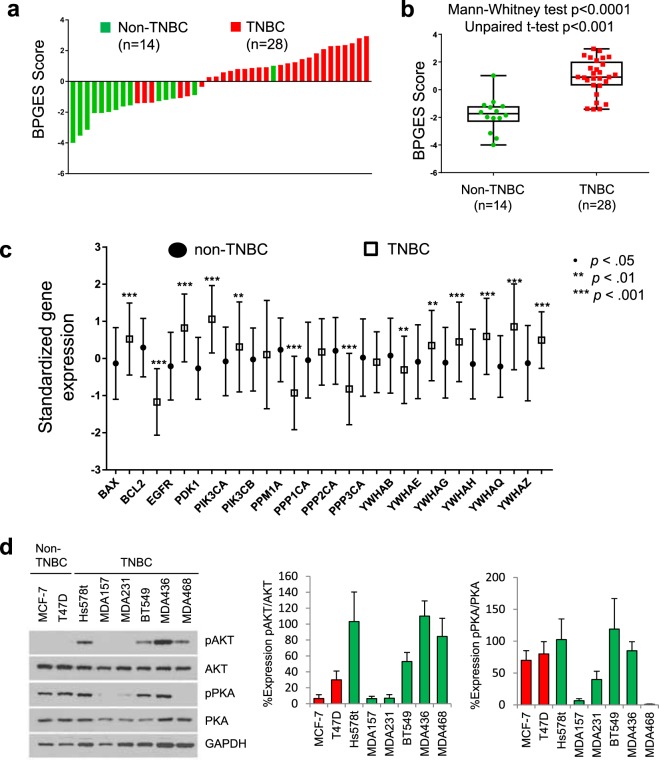
The BPGES was associated with triple-negative status in breast cancer cell lines of the CCLE. **(a)** Waterfall plot showing the BPGES in 42 breast cancer cell lines from the CCLE database (www.broadinstitute.org). The mean BPGES score was −1.81 ± 0.33 in non-TNBC cells (n = 14) versus .90 ± 0.25 in TNBC cells (n = 28) (*p* = 4.22 × 10^−7^). The graph was generated using GraphPad Prism 7. **(b)** Box plots showing differences in the median BPGES score between non-TNBC cell (n = 14) and TNBC cells (n = 28) from the CCLE database. The graph was generated using GraphPad Prism 7. **(c)** Boxplots showing the differential expression of individual genes of the BPGES between non-TNBC (solid circles) and TNBC cell lines (open squares). Unpaired T-test indicates significance of ***p < 0.001, **p < 0.01, *p < 0.05, no symbol: p > 0.05. **(d)** Western blot showing the expression of active (phosphorylated) and total isoforms of the BAD protein kinases, AKT and PKA in a panel of non-TNBC and TNBC cell lines. Bar graphs show the percent expression of phosphorylated (active) protein in relation to total protein for AKT and PKA after densitometry analysis of at least 3 replicate experiments. All proteins were first normalized to the expression of GAPDH. Error bars indicate standard error of the mean.

To determine whether expression of the BAD pathway was associated with triple-negative status in cell lines, the BPGES was evaluated using expression data downloaded from the CCLE database (www.broadinstitute.org). Similar to our results shown in the tumour samples, gene expression data from 42 breast cancer cell lines (28 TNBC, 14 non-TNBC) indicated a differential expression of the BAD pathway in TNBC cell lines, with a mean BPGES score of −1.81 ± 0.33 in non-TNBC cells versus a mean BPGES score of .90 ± 0.25 in TNBC cells (unpaired *t* test, *P* = 4.22 × 10^−7^; Fig. 3A,B). Figure 3c shows the performance of the individual genes of the BPGES in the CCLE dataset. As shown, the performance of BPGES genes in breast cancer cell lines was similar to their performance in tumour samples. Therefore, cell line models were used to determine whether disruption of the BAD pathway via BAD protein kinase inhibitors could enhance the cytotoxic effects of nonspecific chemotherapy. To do this, a panel of randomly selected ER^+^/PR^+^ (MCF-7, T47D) and TNBC (Hs578t, MDA-157, MDA-231, BT549, MDA-436, MDA-468) cell lines were treated with inhibitors of BAD protein kinases. The 5-year survival probability HRs suggested that TNBC patients have a 2-fold increased risk of death if their tumours display increased expression of phospho-BAD-Ser^136^ (AKT phosphorylation site) or phospho-BAD-Ser^155^ (PKA phosphorylation site). As such, we evaluated whether BAD pathway disruption was capable of sensitizing TNBC cells to cisplatin by using the AKT inhibitor perifosine and the PKA inhibitor H89. Figure 3d shows the relative phosphorylated (active)/native protein expression levels of AKT and PKA as determined by densitometry analyses of replicate Western blots.

**Figure 4:**
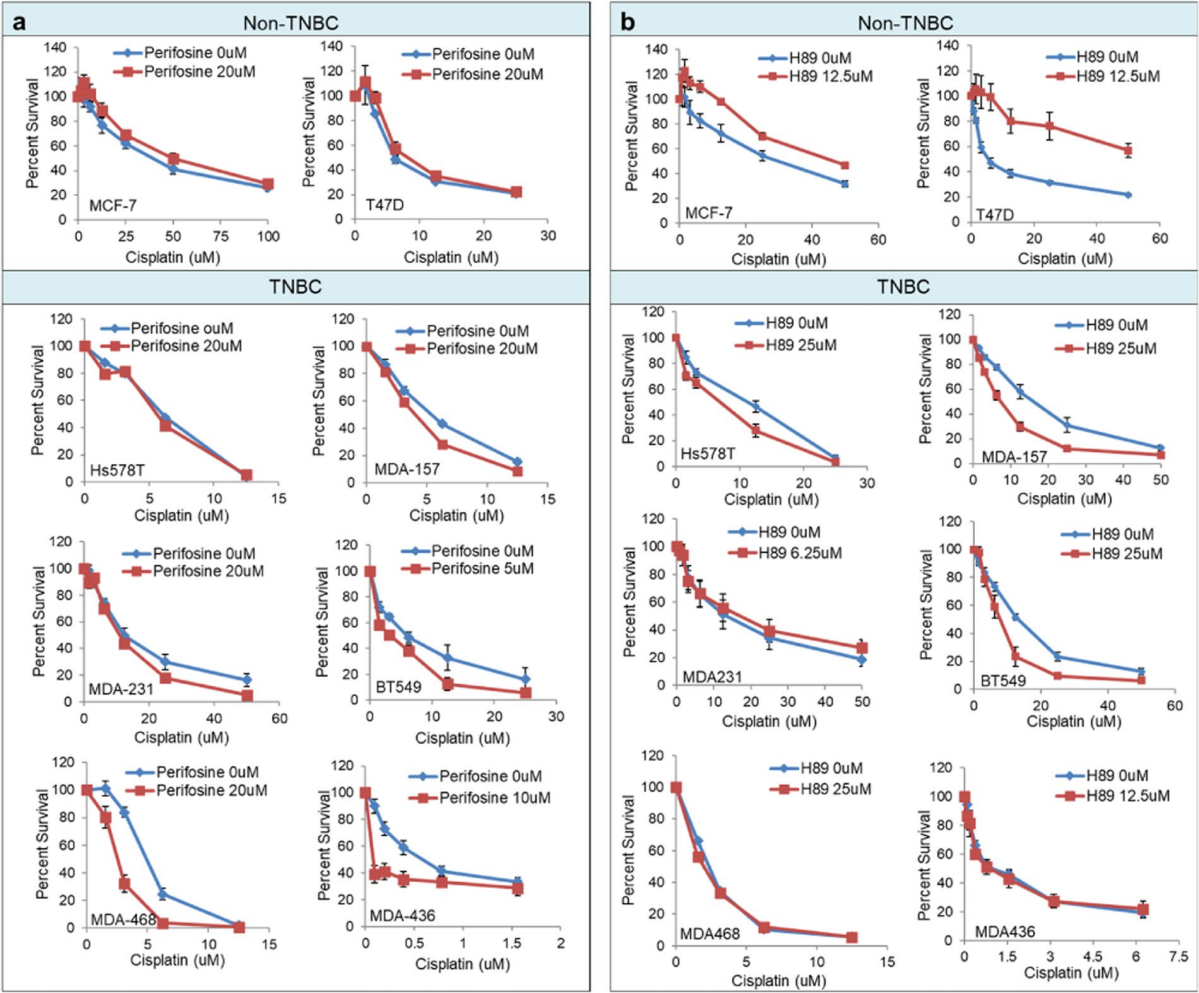
Targeted inhibition of BAD protein kinases increases platinum sensitivity in TNBC cells. CellTiter96, MTS cell survival assays showing the effect of a fixed dose of **(a)** perifosine (AKT inhibitor) and **(b)** H89 dihydrochloride hydrate (H89) on the sensitivity of breast cancer cell lines to cisplatin. Error bars indicate standard error of the mean from 3 replicate experiments.

Data obtained from MTS cell viability assays showed that most TNBC cells treated with a fixed dose of perifosine or H89 were more sensitive to the cytotoxic effects of cisplatin than cells treated with cisplatin alone. In contrast, the sensitivity of non-TNBC cells to cisplatin did not significantly change or was lessened in the presence of perifosine and H89 (Fig. 4a,b). Comparing cisplatin IC_50_ values in the presence and absence of perifosine or H89, which were calculated using the sigmoidal dose-response algorithm (as implemented in GraphPad Prism 7), indicated that the potentiation of cisplatin by a BAD protein kinase inhibitor was significant in the majority of TNBC cells but not in non-TNBC cells (Table 1). The ability of an AKT or H89 inhibitor to enhance cisplatin cytotoxicity depended on the expression of the active phosphorylated isoform of the target kinase in TNBC cells. Treatment of the TNBC cell MDA-468 with perifosine but not H89 potentiated the cytotoxicity of cisplatin. Densitometry analyses of immunoblots suggested that nearly 80% of AKT within this cell line was phosphorylated. In contrast, MDA-468 expressed almost no phosphorylated PKA. However, there were exceptions. In some TNBC cell lines, such as Hs578t, which showed high levels of phosphorylated AKT and low levels of phosphorylated PKA, cisplatin cytotoxicity appeared to be enhanced by H89 but not by perifosine. The reason for this was not explored. However, kinases other than AKT, such as PAK-1 and PKC-iota^54^, have been shown to phosphorylate BAD protein at ser-136 and may have more dominant roles in this cell line.

**Table 1:** TNBC and non-TNBC cell cisplatin IC_50_ values in the presence and absence of the BAD pathway kinase inhibitors perifosine and H89. Drug IC_50_ values were computed using GraphPad with Sigmoidal dose-response algorithm.

Cell line	Log IC_50_		Standard Error		*P* value
**Cisplatin vs cisplatin + perifosine**					
	Perifosine	Perifosine	Perifosine	Perifosine	
	0 μM	5 (or 20) μM	0 μM	5 (or 20) μM	
**Non-TNBC cells**					
MCF-7	1.2730	1.3610	0.0757	0.0545	0.3484
T47D	0.7591	0.8554	0.0450	0.0291	0.0845
**TNBC cells**					
Hs578T	0.7096	0.7664	0.0245	0.0165	0.0592
MDA-157	0.6779	0.5646	0.0141	0.0116	<0.0001
MDA-231	1.0210	1.0110	0.0757	0.0545	0.3484
BT549	0.6101	0.4233	0.0301	0.0429	0.0009
MDA-436	−0.3877	NA	0.0413	NA	NA
MDA-468	0.6690	0.3878	0.0145	0.0199	<0.0001
**Cisplatin vs cisplatin + H89**					
	H89 0 μM	H89 25 (or 12.5) μM	H89 0 μM	H89 25 (or 12.5) μM	
**Non-TNBC cells**					
MCF-7	1.1200	1.2110	0.0763	0.0488	0.3158
T47D	0.5374	1.1640	0.0344	0.1454	0.0008
**TNBC cells**					
HS578T	0.9219	0.7339	0.0323	0.0483	0.0584
MDA-157	1.1180	0.7797	0.0275	0.0207	<0.0001
MDA-231	0.7749	0.8484	0.0817	0.0907	0.5525
BT549	0.9954	0.8362	0.0294	0.0356	0.0020
MDA-436	−0.2467	−0.3447	0.0385	0.0641	0.1936
MDA-468	0.6941	0.9348	0.1401	0.2755	0.4426

**Figure 5:**
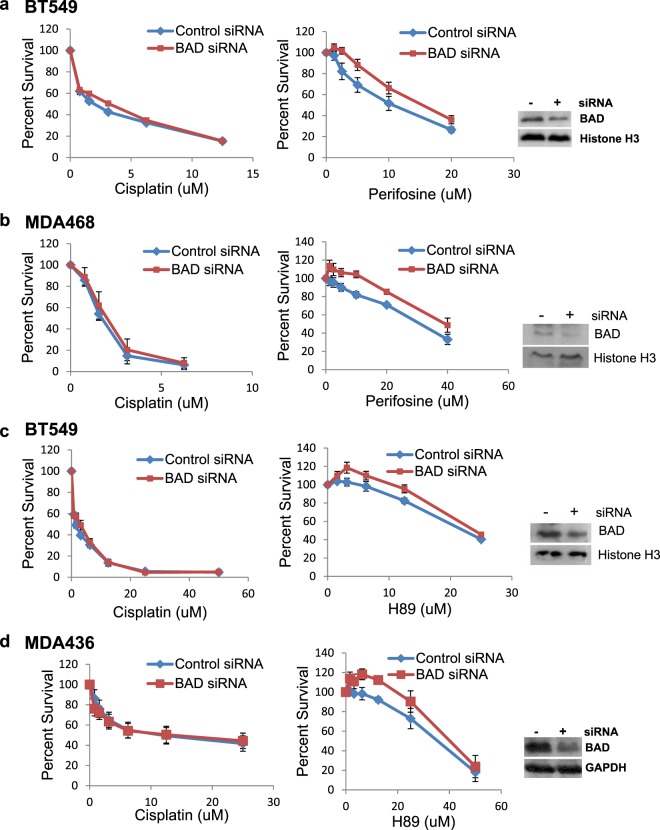
The potentiation of cisplatin by perifosine and H89 was mediated by the functional inhibition of BAD protein. CellTiter96, MTS cell survival assays showing siRNA depletion of BAD protein did not affect the sensitivity of TNBC cells to cisplatin but reduced the sensitivity of **(a)** BT549 and **(b)** MDA468 to the AKT inhibitor, perifosine as well as the sensitivity of **(c)** BT549, and **(d)** MDA436 to the PKA inhibitor, H89. Western blots show depletion of BAD protein by siRNA. Histone H3 and GAPDH were used as loading controls.

In TNBC cell lines, where potentiation of platinum agents was observed, we sought to determine whether the enhancement of cisplatin cytotoxicity was mediated by BAD protein. The TNBC cell lines BT549, MDA468, and MDA436 were depleted of BAD by using siRNA and evaluated for sensitivity to cisplatin, H89, and/or perifosine. As shown, depletion of BAD protein in BT549 (Fig. 5a) and MDA468 (Fig. 5b) did not change cell line sensitivity to cisplatin but resulted in decreased sensitivity to perifosine. Similar results were observed with H89 in BT549 (Fig. 5c) and MDA436 (Fig. 5d) cells. This suggests that BAD protein was required for cisplatin sensitization effects of H89 and perifosine.

## Discussion

Although TNBCs account for only about 17% of all breast cancers^1,8,55^, they are heterogeneous, biologically aggressive, and carry a poorer prognosis than the growth factor receptor–driven or hormone receptor–driven breast cancers^9,10,55^. The earlier age of onset and lethality of TNBC underscores the need for the development of more efficacious therapeutic strategies. Few targeted agents have shown effectiveness against this aggressive form of breast cancer, and the majority of TNBC patients are treated with nonspecific chemotherapeutic agents, including platinums, anthracyclines, and taxanes^7^. Therapies that enhance cancer cell sensitivity to cytotoxic agents could significantly improve patient outcomes.

In this study, we show that the BAD pathway is a targetable biomarker to increase the response of TNBC to nonspecific chemotherapeutic agents, such as cisplatin. PCA was used to develop an expression signature based a subset of genes that comprise the GeneGo Metacore–defined BAD pathway. This BPGES) was derived using 16 genes that showed the most importance within the PCA model, including BAX, BCL-2, EGFR, PDPK1 (PDK1), PIK3CA, PIK3CB (PI3 kinase), PPP1CA (PP1), PPP2CA (PP2A), PPP3CA (Calcineurin), (PPM1A) PP2C, YWHAB, YWHAE, YWHAG, YWHAH, YWHAQ, and YWHAZ (14.3.3). The BPGES was capable of delineating TNBC tumours from hormone responsive (non-TNBC) tumours in independent clinic-genomic breast cancer datasets, including TCC (*n* = 106) and TCGA (*n* = 408) as well as the breast cancer cell lines of the CCLE (*n* = 42). Furthermore, the BPGES was associated with decreased survival probability in both the TCC and TCGA cohorts. TNBC is known to be more aggressive than hormone responsive tumours. Therefore, we evaluated whether the performance of the BPGES was an artifact of increased TNBC proliferation. As described above, although the BPGES showed a correlation with the Hallmark cell cycle signature (r^2^ = 0.54), log-rank tests of Kaplan-Meier curves indicated that cell cycle alone could not explain the association between the BPGES and decreased OS among patients with breast cancer (log-rank *P* = 0.13). All genes within the BPGES have previously been shown to directly or indirectly influence the phosphorylation status and/or apoptotic function of the BAD pathway, including BAX^34,35^, BCL-2, EGFR^36,37^, PDK1 (PDPK1)^38^, PI3 kinase (PIK3CA, PIK3CB)^36^, PP1 (PPP1CA)^39^, PP2A (PPP2CA)^40^, calcineurin (PPP3CA)^41^, (PP2C) PPM1A^25,27,42^, and 14.3.3 (YWHAB, YWHAE, YWHAG, YWHAH, YWHAQ, YWHAZ)^43^. The performance of the individual genes were similar in the TCC and TCGA cohorts and in the CCLE cell lines despite the fact that different gene expression platforms were used for each dataset. Genes encoding 3 of 4 documented BAD protein phosphatases, PP2C^25,27,42^, PP2A^40^, and calcineurin^41^, showed decreased expression, whereas genes encoding the kinases EGFR^36,37^, PDK1^38^, and PI3 kinase^36^ showed increased expression in TNBC compared to non-TNBC cases and cell lines. This suggests an expression profile in TNBC that favoured the phosphorylation and inactivation of BAD protein. In parallel, TNBC cases displayed increased expression of 5 out of 6 genes encoding 14.3.3. compared to non-TNBC cases. 14.3.3 has been reported to inhibit BAD-induced cell death by binding and sequestering BAD when phosphorylated on Ser^136^, a known AKT phosphorylation site^43^. Overall, the performance of the individual genes of the BPGES suggests a prosurvival phenotype in TNBC cases compared to non-TNBC cases. Indeed, immunohistochemistry analyses of a study-generated TMA showed that TNBC cases expressed higher levels of phospho-BAD isoforms, most notably the AKT phosphorylation site BAD-Ser^136^ compared to non-TNBC cases. We next sought to determine whether inhibition of BAD pathway signalling would increase the sensitivity of TNBC cells to a nonspecific chemotherapy agent. HRs of 5-year survival curves suggested that TNBC patients were at 2-fold increased risk of death if their tumours expressed increased levels of phospho-BAD-Ser^136^ or phospho-BAD-Ser^155^. Therefore, we used the AKT inhibitor perifosine and the PKA inhibitor H89. Treating TNBC cells but not non-TNBC cells with perifosine or H89 increased the cytotoxic effects of cisplatin. Although there were exceptions, the ability of the BAD protein kinase inhibitor to enhance cisplatin cytotoxicity appeared to be dependent on the expression of the target kinase. The inhibition of AKT and PKA did not sensitize all TNBC cell lines to cisplatin. Although the reasons for this were not explored, it must be noted that there are several antiapoptotic BAD protein kinases, including PAK1, PKC-iota, ERK, IKK, C-raf, and CK2, that were not evaluated in this study^54^.

These results are consistent with the work of others. It is well known that AKT phosphorylation of BAD protein is a negative regulator of the proapoptotic function of BAD protein^56^. Consistent with our data, other groups have shown that activated AKT signalling is increased in TNBC^57–60^ and that inhibition of AKT signalling can increase the sensitivity of TNBC cells to nonspecific chemotherapies^61^. Others have shown that targeting the pro-survival BCL2 proteins of the BAD pathway, using the BH3 mimetic ABT-737, sensitized basal-like breast cancers to chemotherapy^62^. Although the classifications of basal-like and triple negative are not synonymous, the majority of basal-like breast cancers are triple negative^63^.

Collectively, our results suggest that expression and activation of the BAD pathway may be important mechanisms that TNBC could use to resist therapeutic apoptotic stimuli. Therefore, therapies that enhance TNBC sensitivity to cytotoxic agents could significantly improve patient outcomes. The data presented here suggest that the BAD pathway may be a useful therapeutic target for the treatment of TNBC.

## Supplementary information

Supplementary Information
