# Supplementary material for: Expression of the BAD pathway is a marker of triple-negative status and poor outcome
Source: Sci Rep. 2019 Nov 25;9:17496. doi: 10.1038/s41598-019-53695-0 (PMC6877530; doi:10.1038/s41598-019-53695-0)
Supplement: Supplementary file 1 — Supplementary Information [file 41598_2019_53695_MOESM1_ESM.pdf]

Expression of the BAD pathway is a marker of triple-negative status and poor outcome

Bernadette M. Boac[1,2], Forough Abbasi[3], Roohi Ismail-Khan[4,5], Yin Xiong[1,2], Atif Siddique[1], Hannah Park[1,2], Mingda Han[1,2], Daryoush Saeed-Vafa[1], Hatem Soliman[4,5], Brendon Henry[1], M. Juliana Pena[1], E. Clair McClung[6], Sharon E. Robertson[7], Sarah L. Todd[5], Alex Lopez[1], Weihong Sun[5], Susmitha Apuri[5], Johnathan M. Lancaster[8], Anders E. Berglund[9], Anthony M. Magliocco[10], & Douglas C. Marchion[1,2,*]

[1] Department of Anatomic Pathology, H. Lee Moffitt Cancer Center and Research Institute, Tampa, FL 33612, USA

[2] Chemical Biology and Molecular Medicine, H. Lee Moffitt Cancer Center and Research Institute, Tampa, FL 33612, USA

[3] Cedars-Sinai Medical Center, Los Angeles, CA 90048, USA

[4] Department of Oncologic Sciences, H. Lee Moffitt Cancer Center and Research Institute, Tampa, FL 33612, USA

[5] Department of Women's Oncology, H. Lee Moffitt Cancer Center and Research Institute, Tampa, FL 33612, USA

[6] University of Arizona Cancer Center, Obstetrics and Gynecology, Tucson, AZ 85724, USA

[7] Indiana University Health, Indianapolis, IN 46202, USA

[8] Myriad Genetics, Salt Lake City, UT 84108, USA

[9] Department of Bioinformatics and Biostatistics, H. Lee Moffitt Cancer Center and Research Institute, Tampa, FL 33612, USA

[10] Protean Diagnostics, Tampa, FL 33612, USA

*Corresponding author: dcmarchion@gmail.com

TCC		TCGA		CCLE	
Probe Set ID	gene symbols	TCGA ID 16	gene symbols	probe set Id	gene symbol
merck-NM_138761_s_at	BAX	581	BAX	211833_s_at	BAX
merck-NM_000633_at	BCL2	596	BCL2	203685_at	BCL2
merck2-NM_005228_at	EGFR	1956	EGFR	201983_s_at	EGFR
merck2-L42450_a_at	PDK1	5163	PDK1	226452_at	PDK1
merck-R36545_x_at	PIK3CA	5290	PIK3CA	204369_at	PIK3CA
merck-NM_006219_a_at	PIK3CB	5291	PIK3CB	212688_at	PIK3CB
merck-AK124299_at	PPM1A	5494	PPM1A	203966_s_at	PPM1A
merck-NM_001008709_at	PPP1CA	5499	PPP1CA	200846_s_at	PPP1CA
merck-BC002657_a_at	PPP2CA	5515	PPP2CA	208652_at	PPP2CA
merck2-BQ431179_a_at	PPP3CA	5530	PPP3CA	202429_s_at	PPP3CA
merck-NM_003404_s_at	YWHAB	7529	YWHAB	217718_s_at	YWHAB
merck2-BM755263_a_at	YWHAE	7531	YWHAE	213655_at	YWHAE
merck2-DB483456_at	YWHAG	7532	YWHAG	222985_at	YWHAG
merck2-X78138_at	YWHAH	7533	YWHAH	201020_at	YWHAH
merck2-BQ925373_x_at	YWHAQ	10971	YWHAQ	213699_s_at	YWHAQ
merck2-BC101483_a_at	YWHAZ	7534	YWHAZ	200640_at	YWHAZ

Supplementary Table 1. Gene IDs and gene symbols of the BAD pathway gene expression signature for the Moffitt dataset (Affymetrix HuRSTA gene chip), The Cancer Genome Atlas (TCGA, RNAseq), and The Cancer Cell Line Emporium (CCLE, Affymetrix U133Plus 2.0 gene chip). For the Moffitt and CCLE datasets, single probe sets were selected on the basis of the greatest expression value. Moffitt data are available on the Gene Expression Omnibus (GEO) website, GEO accession #GSE62931.

Supplementary Table S2. Pathologist scoring of immunohistochemistry stains for pBAD-ser112, pBAD-ser136, pBAD-ser155, and pAKT. Scores are a product of cellularity (0-3) and intensity (0-3).

**non-TNBC Samples**					
**Sample #**	**Core ID**	**pBAD 112**	**pBAD S136**	**pBAD 155**	**pAKT**
**non-TNBC-41**	21	0	2	2	0
	112	1	2	1	0
	244	0	2	2	0
**non-TNBC-15**	34	1	2	1	0
	124	4	4	3	1
	210	3	2	3	0
**non-TNBC-08**	2	1	6	2	0
	83	2	2	1	0
	222	4	3	2	0
**non-TNBC-51**	9	3	3	3	0
	131	6	6	6	1
	198	6	4	9	2
**non-TNBC-14**	18	3	3	6	0
	142	4	3	2	0
	229	2	3	3	0
**non-TNBC-12**	5	3	3	3	0
	128	3	4	6	0
	215	6	2	9	0
**non-TNBC-27**	77	3	3	3	0
	162	4	3	2	0
	207	6	6	6	0
**non-TNBC-25**	50	3	6	2	2
	153	n/a	3	n/a	0
	225	3	6	4	0
**non-TNBC-03**	70	2	3	2	0
	146	2	2	3	0
	216	9	6	3	3
**non-TNBC-45**	36	3	6	2	0
	150	3	2	2	0
	183	3	2	2	0
**non-TNBC-30**	65	n/a	n/a	n/a	n/a
	122	4	3	2	0
	235	3	2	6	0
**non-TNBC-23**	53	3	6	3	1
	144	3	3	6	0
	241	n/a	n/a	3	0
**non-TNBC-50**	59	6	9	9	3
	166	6	6	6	0
	200	3	3	2	2
**non-TNBC-35**	44	2	4	6	0
	110	3	3	3	0
	254	6	6	9	0
**non-TNBC-33**	94	2	6	4	0
	192	4	3	3	0
	248	n/a	n/a	n/a	n/a

Supplementary Table S2 Continued.

**non-TNBC-34**	88	0	2	0	0
	101	0	4	1	0
	194	0	2	0	0
**non-TNBC-52**	68	6	9	3	2
	99	3	4	9	0
	180	6	4	9	0
**non-TNBC-53**	47	6	3	4	0
	91	6	4	4	3
	138	2	n/a	2	0
**TNBC Samples**					
**Core Position**	**Core ID**	**pBAD 112**	**pBAD S136**	**pBAD 155**	**pAKT**
**TNBC-31**	17	0	2	0	0
	84	2	2	2	0
	247	2	2	2	0
**TNBC-05**	52	2	6	3	0
	115	3	4	2	0
	165	2	4	4	0
**TNBC-42**	1	3	6	3	0
	96	2	9	2	1
	187	3	9	6	2
**TNBC-14**	75	4	3	2	2
	148	2	3	4	0
	219	3	6	2	2
**TNBC-40**	11	6	6	9	0
	82	4	3	4	0
	158	4	3	9	0

Supplementary Table S2 Continued.

**TNBC-39**	33	2	2	3	0
	103	2	3	2	0
	202	3	2	6	0
TNBC-04	19	2	6	3	6
	145	0	4	2	3
	199	2	3	2	3
TNBC-38	46	2	9	4	2
	139	2	6	4	3
	193	2	9	3	6
TNBC-11	30	3	3	2	0
	104	6	3	2	0
	171	6	3	2	0
TNBC-46	3	6	3	6	0
	129	2	3	2	0
	255	9	6	9	0
TNBC-47	76	4	3	2	2
	120	3	6	6	2
	223	2	4	4	2
TNBC-16	95	6	2	3	0
	169	2	3	4	0
	189	2	2	2	0
TNBC-22	60	6	6	9	2
	118	3	4	4	0
	176	3	6	4	2
TNBC-41	37	2	6	2	0
	109	6	3	6	0
	209	2	6	6	3
TNBC-48	32	3	6	4	2
	86	4	3	6	0
	170	3	6	4	0
TNBC-10	6	4	6	9	0
	92	9	6	6	2
	234	6	6	6	2
TNBC-44	25	0	3	0	0
	114	0	2	0	0
	190	0	3	0	0
TNBC-15	73	2	n/a	9	n/a
	123	4	2	3	0
	212	4	6	3	2
TNBC-09	48	3	3	4	0
	141	2	3	2	0
	242	2	3	2	0
TNBC-12	10	2	3	2	1
	78	4	6	6	2
	173	3	6	3	2

Supplementary Table S2 Continued.

TNBC-02	14	3	9	3	3
	143	n/a	n/a	n/a	0
	231	n/a	n/a	n/a	n/a
TNBC-03	69	6	9	9	0
	127	3	4	4	0
	178	n/a	n/a	n/a	n/a
TNBC-26	137	n/a	n/a	n/a	n/a
	184	n/a	n/a	n/a	n/a
	230	6	3	3	n/a
TNBC-06	45	n/a	n/a	n/a	n/a
	152	4	3	6	2
	179	3	3	3	0
TNBC-17	57	6	3	3	0
	185	2	2	6	0
	233	n/a	n/a	n/a	n/a
TNBC-27	20	3	9	6	0
	147	6	6	9	0
	228	9	3	9	0
TNBC-28	29	2	4	3	0
	90	2	2	3	0
	177	6	4	4	0
TNBC-20	39	6	6	3	2
	149	2	4	4	0
	217	6	6	6	2
TNBC-25	66	2	6	2	0
	157	2	3	2	0
	224	n/a	3	n/a	n/a
TNBC-36	4	9	6	6	0
	140	9	6	6	2
	246	n/a	n/a	9	2
TNBC-32	26	6	9	6	6
	108	9	9	9	6
	174	9	9	9	6
TNBC-01	7	3	6	3	3
	100	2	4	3	2
	159	2	n/a	n/a	2
TNBC-37	49	3	6	2	2
	111	2	6	2	2
	245	3	6	3	2
TNBC-21	72	n/a	n/a	n/a	n/a
	133	6	9	6	6
	227	9	6	6	3
TNBC-35	58	6	9	6	3
	161	4	3	2	0
	208	4	6	4	2
TNBC-51	28	2	6	3	0
	61	2	4	3	0
	240	n/a	n/a	n/a	0

Supplementary Table S3. Definiens software analysis of immunohistochemistry stains for pBAD-ser112, pBAD-ser136, pBAD-ser155, and pAKT.

**non-TNBC Sampels**				
**Sampel #**	Core ID	BAD112	BAD136	BAD155
**non-TNBC-41**	21	7	32	15
	112	6	31	12
	244	11	23	31
**non-TNBC-15**	34	21	30	4
	124	89	45	29
	210	83	n/a	28
**non-TNBC-08**	2	91	41	29
	83	112	n/a	37
	222	110	50	50
**non-TNBC-51**	9	n/a	88	94
	131	165	127	135
	198	112	75	91
**non-TNBC-14**	18	n/a	38	45
	142	155	n/a	99
	229	n/a	n/a	n/a
**non-TNBC-12**	5	144	69	86
	128	121	58	132
	215	169	21	141
**non-TNBC-27**	77	125	126	87
	162	116	97	75
	207	126	115	115
**non-TNBC-25**	50	142	102	65
	153	n/a	n/a	n/a
	225	137	85	84
**non-TNBC-03**	70	77	103	39
	146	68	82	69
	216	151	133	120
**non-TNBC-45**	36	136	107	45
	150	83	63	37
	183	116	69	64
**non-TNBC-30**	65	n/a	n/a	n/a
	122	87	43	34
	235	115	74	103
**non-TNBC-23**	53	99	144	74
	144	68	97	111
	241	n/a	n/a	55
**non-TNBC-50**	59	115	136	83
	166	127	141	135
	200	69	120	42
**non-TNBC-35**	44	98	60	85
	110	104	49	85
	254	127	71	134
**non-TNBC-33**	94	109	141	79
	192	77	80	65
	248	n/a	n/a	n/a

Supplementary Table S3 continued.

**non-TNBC-34**	88	7	41	9
	101	9	49	8
	194	7	45	7
**non-TNBC-52**	68	129	178	74
	99	138	91	164
	180	124	75	169
**non-TNBC-53**	47	129	80	66
	91	152	103	81
	138	n/a	n/a	n/a
**TNBC Samples**				
**Sampel #**	Core ID	BAD112	BAD136	BAD155
**TNBC-31**	17	8	49	1
	84	49	48	22
	247	66	94	48
**TNBC-05**	52	89	62	76
	115	64	88	52
	165	72	70	70
**TNBC-42**	1	145	44	90
	96	103	106	33
	187	112	99	52
**TNBC-14**	75	107	n/a	62
	148	91	82	67
	219	122	139	79
**TNBC-40**	11	178	171	197
	82	84	104	98
	158	101	109	144

Supplementary Table S3 continued.

**TNBC-39**	33	n/a	n/a	n/a
	103	90	n/a	39
	202	78	48	73
**TNBC-04**	19	47	112	30
	145	8	72	18
	199	41	98	32
**TNBC-38**	46	33	162	34
	139	49	158	80
	193	65	176	62
**TNBC-11**	30	147	n/a	76
	104	97	126	29
	171	125	104	45
**TNBC-46**	3	174	107	178
	129	137	54	83
	255	166	138	159
**TNBC-47**	76	95	114	72
	120	121	79	93
	223	54	83	48
**TNBC-16**	95	145	34	90
	169	70	97	102
	189	19	52	31
**TNBC-22**	60	147	167	118
	118	74	104	61
	176	126	153	96
**TNBC-41**	37	80	97	55
	109	102	82	55
	209	32	130	67
**TNBC-48**	32	n/a	n/a	n/a
	86	104	n/a	57
	170	126	88	58
**TNBC-10**	6	158	101	148
	92	176	161	175
	234	71	127	67
**TNBC-44**	25	6	54	1
	114	4	65	3
	190	5	69	1
**TNBC-15**	73	81	n/a	99
	123	68	20	17
	212	n/a	44	29
**TNBC-09**	48	118	94	64
	141	139	53	88
	242	89	39	54
**TNBC-12**	10	74	124	66
	78	122	134	124
	173	121	134	99
**TNBC-02**	14	153	155	128
	143	n/a	n/a	n/a
	231	n/a	n/a	n/a

Supplementary Table S3 continued.

**TNBC-03**	69	124	171	127
	127	121	59	68
	178	n/a	n/a	n/a
**TNBC-26**	137	n/a	n/a	n/a
	184	n/a	n/a	n/a
	230	145	110	n/a
**TNBC-06**	45	n/a	n/a	n/a
	152	88	107	85
	179	126	92	95
**TNBC-17**	57	132	117	80
	185	57	43	76
	233	n/a	n/a	n/a
**TNBC-27**	20	127	64	69
	147	125	57	78
	228	109	84	118
**TNBC-28**	29	112	62	95
	90	109	62	65
	177	125	76	86
**TNBC-20**	39	131	151	100
	149	86	61	54
	217	119	158	122
**TNBC-25**	66	74	142	23
	157	40	117	7
	224	n/a	n/a	n/a
**TNBC-36**	4	209	115	152
	140	179	138	163
	246	n/a	n/a	174
**TNBC-32**	26	173	200	173
	108	168	201	171
	174	177	199	168
**TNBC-01**	7	149	120	117
	100	68	96	64
	159	n/a	n/a	n/a
**TNBC-37**	49	102	178	68
	111	41	148	40
	245	104	158	89
**TNBC-21**	72	n/a	n/a	n/a
	133	135	217	127
	227	177	183	164
**TNBC-35**	58	163	96	111
	161	149	66	116
	208	153	98	108
**TNBC-51**	28	88	166	119
	61	74	43	60
	240	n/a	n/a	n/a

Supplementary Figure S1

a

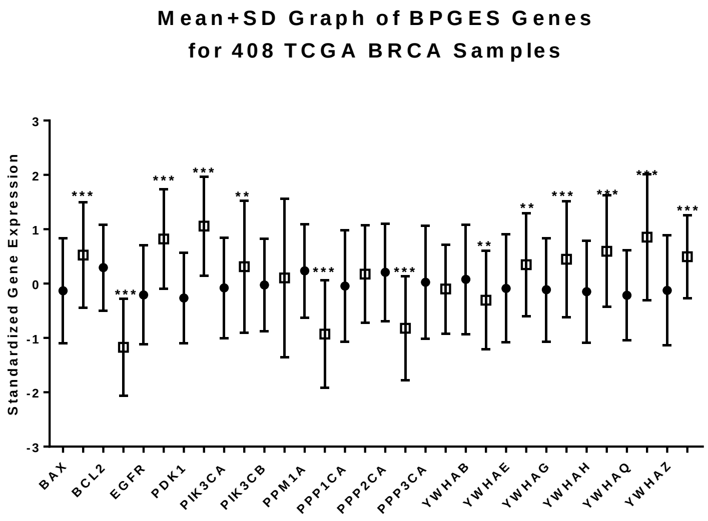

b

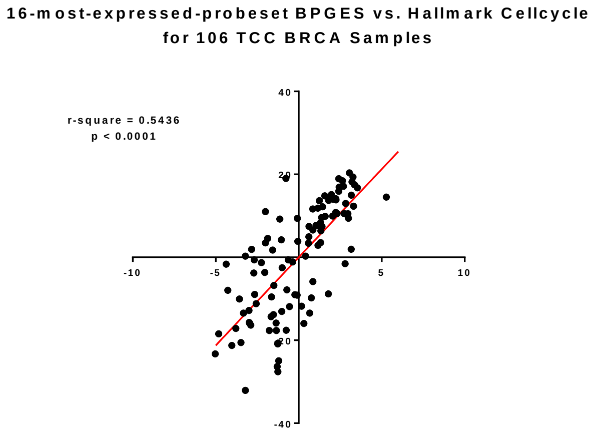

c

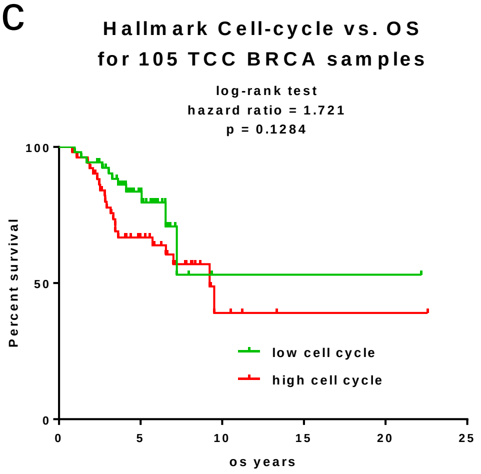

a. Performance of the BPGES genes in the TCGA dataset. Boxplots showing the differential expression of individual genes of the BPGES between non-TNBC (n = 324) and TNBC (n = 84) cases. Unpaired *t* test indicates significance of ***p < .0001, ** p< .001, *p<.05, no symbol: p>.05. **b.** The BPGES is moderately correlated with a cell cycle signature from the Hallmark gene sets. **c.** The Hallmark cell cycle signature was not associated with overall survival in the TCC105 cohort. These data suggest that although cell cycle is an aspect of the BPGES it cannot explain the association between the BPGES and overall survival.

Map of the tissue microarray. Empty cells are used for slide orientation. Numbers in cells signify core IDs.

	0	1.3	2.6	3.9	5.2	6.5	7.8	9.1	10.4	11.7	13	14.3	15.6
**0.0**	Empty	Empty	36	75	76	115	116	155	156	195	196	235	236
**1.3**	Empty	Empty	37	74	77	114	117	154	157	194	197	234	237
**2.6**	Empty	35	38	73	78	113	118	153	158	193	198	233	238
**3.9**	1	34	39	72	79	112	119	152	159	192	199	232	239
**5.2**	2	33	40	71	80	111	120	151	160	191	200	231	240
**6.5**	3	32	41	70	81	110	121	150	161	190	201	230	241
**7.8**	4	31	42	69	82	109	122	149	162	189	202	229	242
**9.1**	5	30	43	68	83	108	123	148	163	188	203	228	243
**10.4**	6	29	44	67	84	107	124	147	164	187	204	227	244
**11.7**	7	28	45	66	85	106	125	146	165	186	205	226	245
**13.0**	8	27	46	65	86	105	126	145	166	185	206	225	246
**14.3**	9	26	47	64	87	104	127	144	167	184	207	224	247
**15.6**	10	25	48	63	88	103	128	143	168	183	208	223	248
**16.9**	11	24	49	62	89	102	129	142	169	182	209	222	249
**18.2**	12	23	50	61	90	101	130	141	170	181	210	221	250
**19.5**	13	22	51	60	91	100	131	140	171	180	211	220	251
**20.8**	14	21	52	59	92	99	132	139	172	179	212	219	252
**22.1**	15	20	53	58	93	98	133	138	173	178	213	218	253
**23.4**	16	19	54	57	94	97	134	137	174	177	214	217	254
**24.7**	17	18	55	56	95	96	135	136	175	176	215	216	255

Supplementary figure S2. Map of the tissue microarray.

Table showing breast cancer sample core location on the tissue microarray. Cells show core IDs for each unique tissue sample. Empty cells are used for slide orientation. X and Y axis are used as coordinates for each core ID.

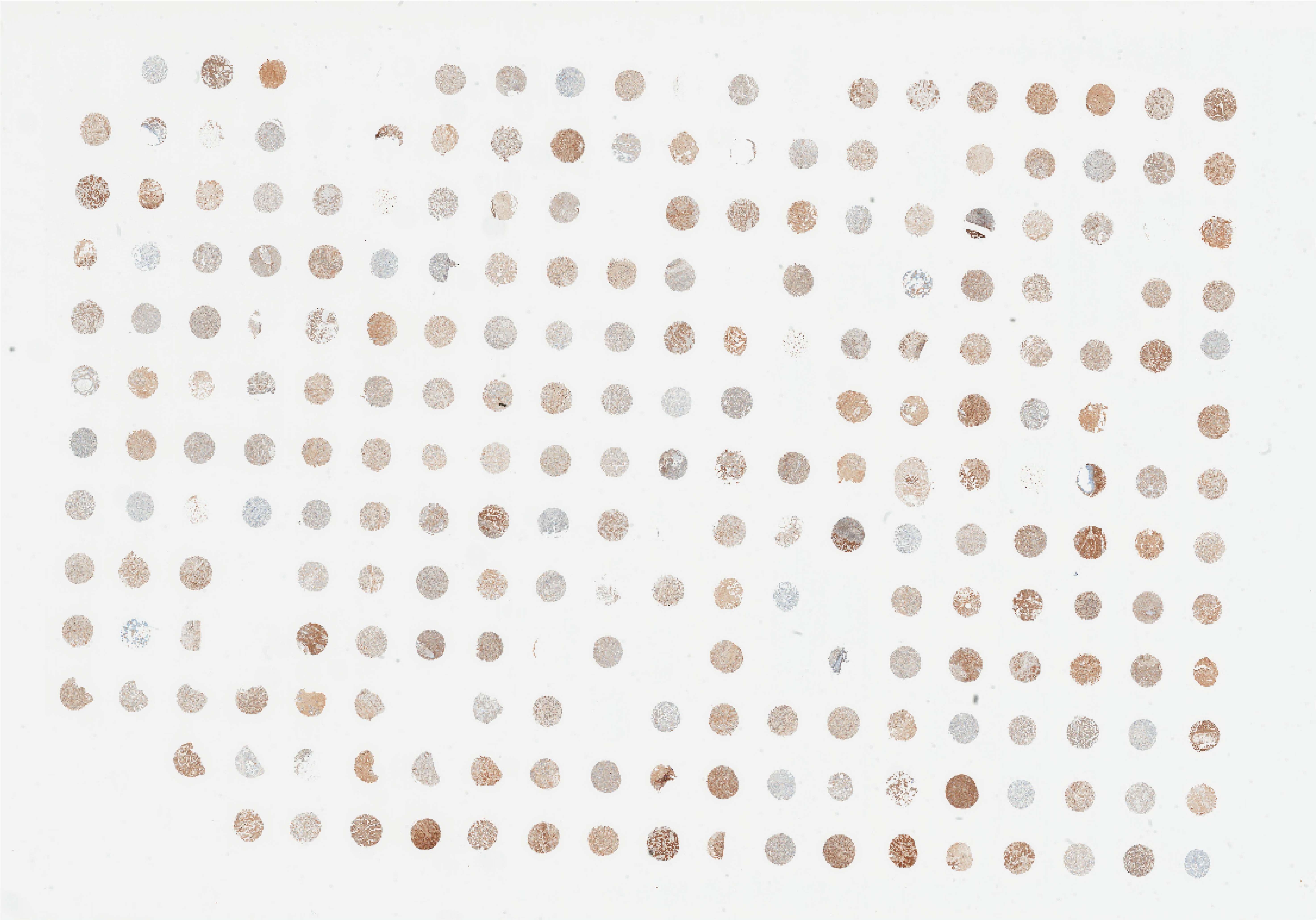

Supplementary figure S3. Phospho-BAD-ser112 levels in breast cancer samples.
A tissue microarray composed of 43 TNBC and 25 non-TNBC samples was evaluated for the expression of phospho-BAD-ser112 by immunohistochemistry.

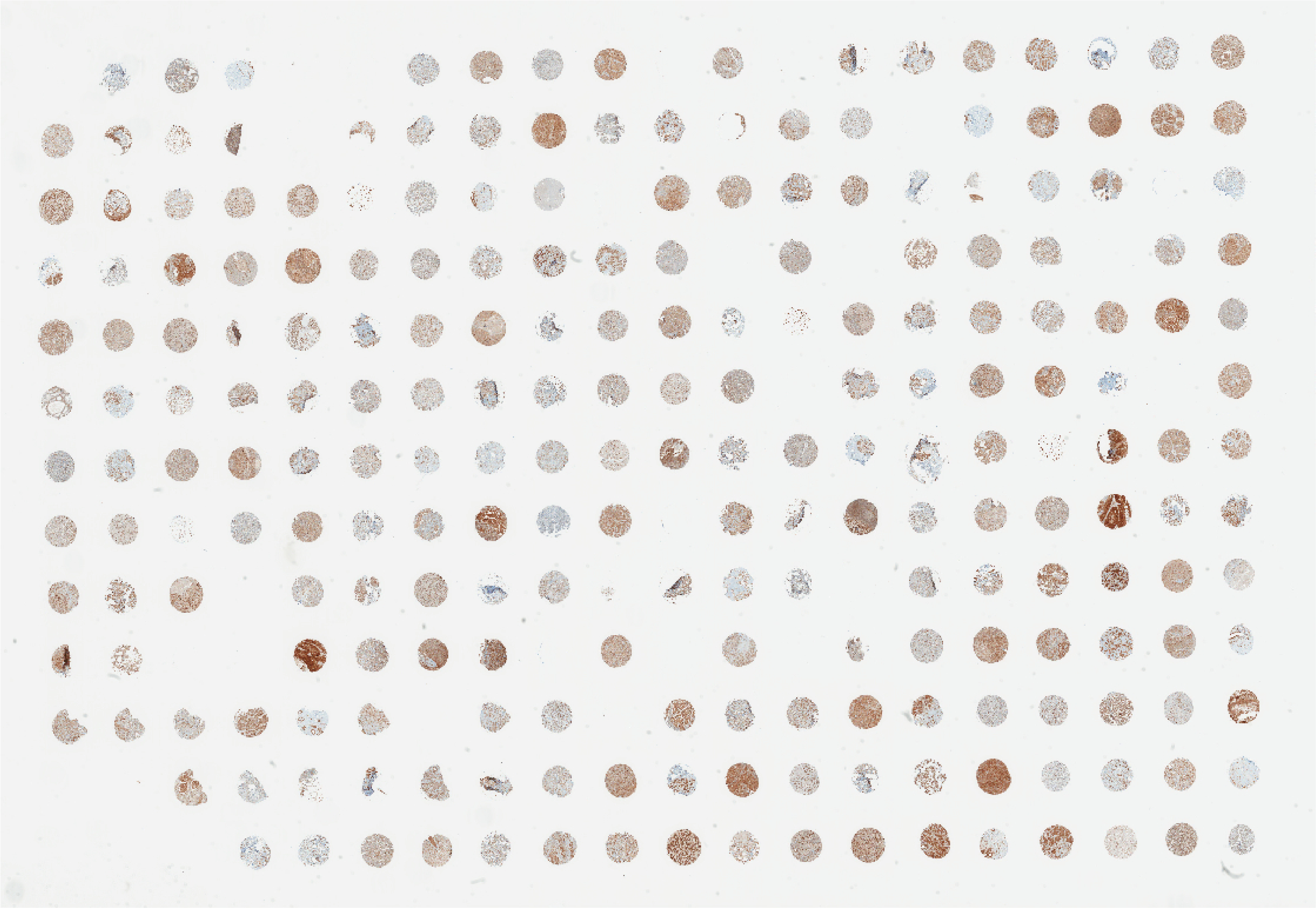

Supplementary Figure S4. Phospho-BAD-ser136 levels in breast cancer samples.
A tissue microarray composed of 43 TNBC and 25 non-TNBC samples was evaluated for the expression of phospho-BAD-ser136 by immunohistochemistry.

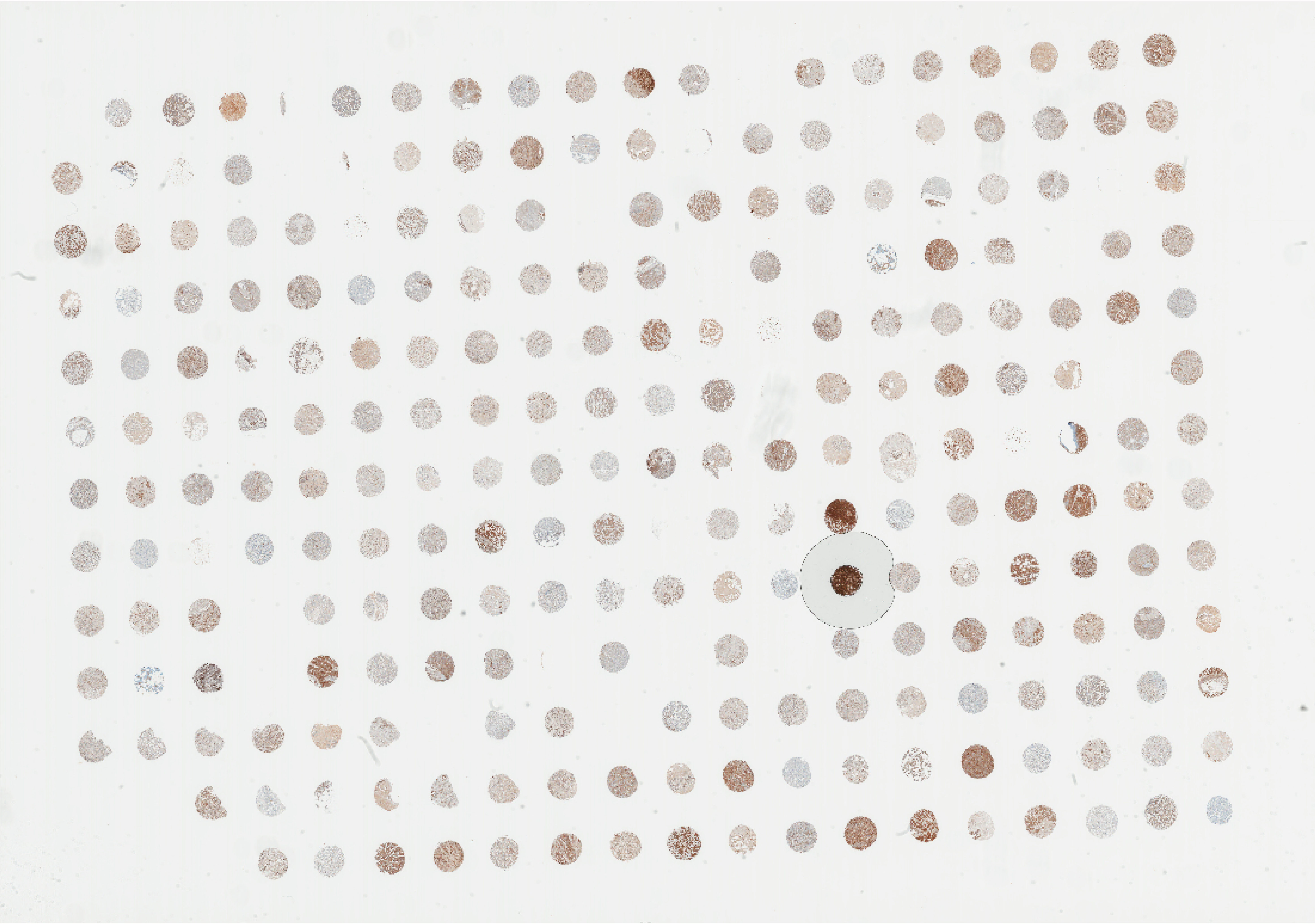

Supplementary figure S5. Phospho-BAD-ser155 levels in breast cancer samples.
A tissue microarray composed of 43 TNBC and 25 non-TNBC samples was evaluated for the expression of phospho-BAD-ser155 by immunohistochemistry.

Supplementary Figure 6.

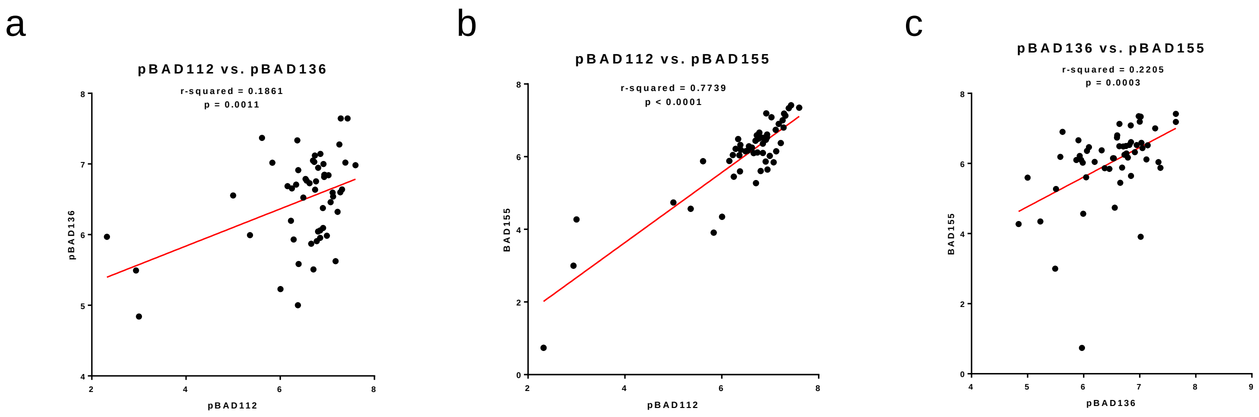

Correlations between phosphorylated BAD (pBAD) isoforms based on Definiens expression scores of immunohistochemistry stains. Data are available for 36 TNBC samples and 18 non-TNBC samples (54 cases in total). **a.** A weak correlation was observed in the expression between pBAD-112 and pBAD-136. **b**. A strong correlation was observed between pBAD-112 and pBAD-155 expression scores. **c.** A moderate correlation was observed between pBAD-136 and pBAD-155 expression scores.
